# Environmental surveillance of fungi and susceptibility to antifungal agents in tertiary care hospitals

**DOI:** 10.1128/spectrum.02270-23

**Published:** 2023-12-04

**Authors:** Parisa Badiee, Abdolkarim Ghadimi-Moghadam, Habibeh Bayatmanesh, Jafar Soltani, Ali Reza Salimi-Khorashad, Fatemeh Ghasemi, Maneli Amin Shahidi, Hadis Jafarian

**Affiliations:** 1 Professor Alborzi Clinical Microbiology Research Center, Shiraz University of Medical Sciences, Shiraz, Iran; 2 Department of Pediatric Infectious Diseases, Emmam Sajjad Hospital, Yasuj University of Medical Sciences, Yasuj, Iran; 3 Department of Pediatrics, Faculty of Medicine, Kurdestan University of Medical Sciences, Sanandaj, Iran; 4 Department of Parasitology and Mycology, School of Medicine, Infectious Diseases and Tropical Medicine Research Center, Zahedan University of Medical Sciences, Zahedan, Iran; NHLS Tygerberg/Stellenbosch University, Cape Town, Western Cape, South Africa

**Keywords:** environmental fungi, *Aspergillus*, *Candida*, *Mucorales*, nosocomial infection

## Abstract

This study aimed to evaluate the epidemiology of fungi in environment of tertiary care hospitals and their susceptibility patterns to seven antifungal agents for controlling and preventing fungal infections in patients. Environmental samplings on the surfaces of different parts of hospitals were collected from four university-affiliated teaching hospitals in Iran. Routine and molecular procedures identified isolated fungi, and antifungal susceptibility tests were performed *in vitro*. Of the 400 samples, 152 presented positive culture results, and 193 fungal species were isolated. Mold fungi accounted for 59.1% (115 species) of isolates. Most isolated fungal species in descending order were 22 *Aspergillus flavus* (11.4%), 21 *Candida albicans* (10.9%), 17 *Mucor* species (8.8%), 16 *Penicillium* species (8.3%), 16 *Candida famata* (8.3%), 15 *Alternaria* species (7.8%), 13 *Fusarium* species (6.7%), 11 *Candida parapsilosis* (5.7%), and 10 *Aspergillus niger sensu stricto* (5.2%). The isolated *Aspergillus* species revealed relatively low susceptibility to itraconazole and posaconazole with MIC90 values of 8 µg/mL. The MIC90 values of amphotericin and luliconazole in *Candida* species were 0.5 and 0.064 µg/mL, respectively. In this study, different species of filamentous and yeast fungi with varying susceptibility to antifungal agents were reported from the environment of the studied hospitals.

**IMPORTANCE:**

Saprophytic fungi can cause nosocomial infections in high-risk patients. These infections are related to high mortality and cost. In the current study, different species of filamentous fungi and yeast were isolated from the environment of the studied hospitals. Some species were resistant to antifungal drugs. We suggest that the future work concentrates on the relationship between the level/quantification of saprophytic contamination in the environment of hospitals and fungal infections in patients.

## INTRODUCTION

Saprophytic fungi like *Mucorales*, *Aspergillus*, *Candida*, and *Fusarium* species can cause outbreaks of nosocomial infections in high-risk patients by transferring from the hospital environment (1). Fungal infections have been on the rise due to the increased number of immunocompromised patients, such as allogeneic hematopoietic stem cell transplantation recipients, solid organ transplantation, the increasing use of invasive devices (central venous catheters), and newer immunomodulatory agents (2 – 4). In the United States, *Aspergillus* (A), *Candida* (C), and *Pneumocystis* accounted for >80% of fungal infections diagnosed in hospitalized patients (5). Exposure to *Aspergillus* spores in hospitals can increase nosocomial aspergillosis in immunocompromised patients (4). In patients with hematologic disorders like leukemia, lung cancer, and non-Hodgkin lymphoma, the mortality rate of aspergillosis was reported to be 13–21% (5). Furthermore, candidiasis was diagnosed in 5% of cancer patients with a 12% mortality rate (5). Nosocomial candidiasis in immunocompromised patients is associated with prolonged hospitalization. The frequency of *Candida* non-*albicans* species causing nosocomial infections has increased over the past few decades, which have been more resistant to treatment than *Candida albicans* (6). Weiner et al. reported that *Candida* species were considered as the fourth most common pathogen across all healthcare-associated infections (7).

There are reports of the isolation of different species of fungi from hospital environments like floors, beds, countertops, baths, and other surfaces (1, 4). In the present research, the epidemiology of fungi in different parts of hospitals and their susceptibility patterns were investigated, aiming at controlling and preventing fungal infections in high-risk patients.

## MATERIALS AND METHODS

### Sites and sampling

The study was a prospective observational study, performed from May 2020 to May 2022 in four university-affiliated teaching hospitals in Iran (Shiraz, Yasuj, Sanandaj, and Zahedan). Different departments with high-risk patients [solid organ transplantation, hematology, pediatric oncology, and intensive care unit (ICU) wards] were selected as sampling sites. The environmental samples were collected from sink faucets and/or traps, around the beds of patients, air-conditioning units, nursing trolleys, tube racks, and ventilators by pre-moistened cotton-tipped swabs. A total of 400 samples (100 from each hospital) were collected and inoculated on the plates containing sabouraud dextrose agar (Merck, Darmstadt, Germany) with 50 mg/mL of chloramphenicol (Merck, Darmstadt, Germany).

### Fungal identification

All the plates were incubated at 24°C for 7 days. To ensure the purity of the isolated fungi, isolated colonies were re-cultured onto separate sabouraud dextrose agar or potato dextrose agar (Merck, Darmstadt, Germany) plates. Isolated fungi were identified based on colony morphology and microscopic evaluation by lactophenol cotton blue and molecular methods.

### DNA extraction

DNA was extracted from yeast species using the lithium acetate–SDS (Sigma, St. Louis, MO, USA) solution (200 mM LiOAc and 1% SDS) and 96% ethanol (Merck, Darmstadt, Germany) according to Lõoke et al. (8). DNA was extracted from young filamentous fungi using the phenol-chloroform method (9). In doing so, isolated mold was grown on sabouraud dextrose broth (Merck, Darmstadt, Germany) and incubated with a shaker (120 rpm) for two (*Aspergillus* species) to three (slow grower molds species) days at 30°C.

### Molecular identification

For the identification of yeast species, ITS1 (5′-TCCGTAGGTGAACCTGCGG-3′) and ITS4 (5′-TCCTCCGCTTATTGATATGC-3′) primers were used as per Mirhendi et al. (10). The PCR products were digested with the *Msp*I (Thermo Fisher Scientific, Vilnius, Lithuania) restriction enzyme. The amplification of filamentous fungi was performed using beta-tubulin gene forward 5′-GGTAACCAAATCGGTGCTGCTTTC-3′ and reverse 5′-ACCCTCAGTGTGACCCTTGGC-3′ primers (9). The PCR products were digested by the *Alw*I (Thermo Fisher Scientific, Vilnius, Lithuania) restriction enzyme. The lengths of amplified and restriction fragment products and the 50-bp DNA ladder (Sinaclon, Tehran, Iran) were visualized by electrophoresis after running in 1.5% and 2% agarose gels (CinnaGen, Tehran, Iran), respectively, for an hour. *Mucorales* were identified as isolates through amplification of the D1/D2 region and subsequent sequencing (11). The PCR products of some isolates were identified by sequencing. The obtained data were compared to the NCBI nucleotide database (BLAST; https://blast.ncbi.nlm.nih.gov/Blast.cgi) and deposited in GenBank. Sequences were edited and manually adjusted in MEGA-X software version 11.0.13 (12). A phylogenetic tree was created using the unweighted pair group method with an arithmetic mean algorithm in the same software. The evolutionary distances were computed using the p-distance method, and the bootstrap analyses were run for 1,000 replicates.

### Antifungal susceptibility testing

The antifungal susceptibility tests of *Candida* species to amphotericin B (AMB), fluconazole (FLU), itraconazole (ITR), voriconazole (VRC), posaconazole (POS), luliconazole (LUL), isavuconazole (ISA), and caspofungin (CAS) were performed according to the microdilution Clinical and Laboratory Standards Institute (CLSI) M27, M59, and M60 methods (13 – 15). Antifungal susceptibility of filamentous fungi to AMB, CAS, VOR, ITR, POS, LUL, and ISA was performed, according to CLSI M38-A2 and M61 documents (16, 17). The powders of VRC, FLU, ITR, and CAS were obtained from Sigma (Sigma, St. Louis, MO, USA), and those of POS and AMB were from Sigma (Sigma, Germany). The concentration ranges of VRC, FLU, POS, ITR, CAS, and AMB were 0.03–16 µg/mL, and those for LUL and ISA were 0.008–8 µg/mL. *Candida parapsilosis* ATCC 22019 were used as quality controls in the same procedure. Antifungal-free well (positive control) and well without fungus (negative control) were included in each row. The 96-well microdilution plates were incubated at 35°C and read after the time procedure presented for yeasts and molds according to CLSI criteria. The concentration of CAS in filamentous isolates causing visible changes in the morphological structure of the hyphae (round, compact, and branched hyphae) was defined as the minimum effective concentration (MEC). The minimum inhibitory concentration (MIC) endpoint for AMB in both yeasts and mold isolates was the lowest concentration inhibiting visible fungal growth (100% inhibition). The MIC values of azole antifungal agents in mold isolates were the lowest concentration inhibiting visible fungal growth (100% inhibition), and that for yeasts species was the lowest concentration inhibiting visible fungal growth (50% inhibition) compared to positive control well growth.

### Statistical analysis

Data of the MIC values were collected in SPSS version 16 (International Business Machines Corp., USA). The MIC/MEC ranges, MIC/MEC50 and MIC/MEC90, and geometrics means (MICGM) for each isolate were calculated.

## RESULTS

**Fig 1:**
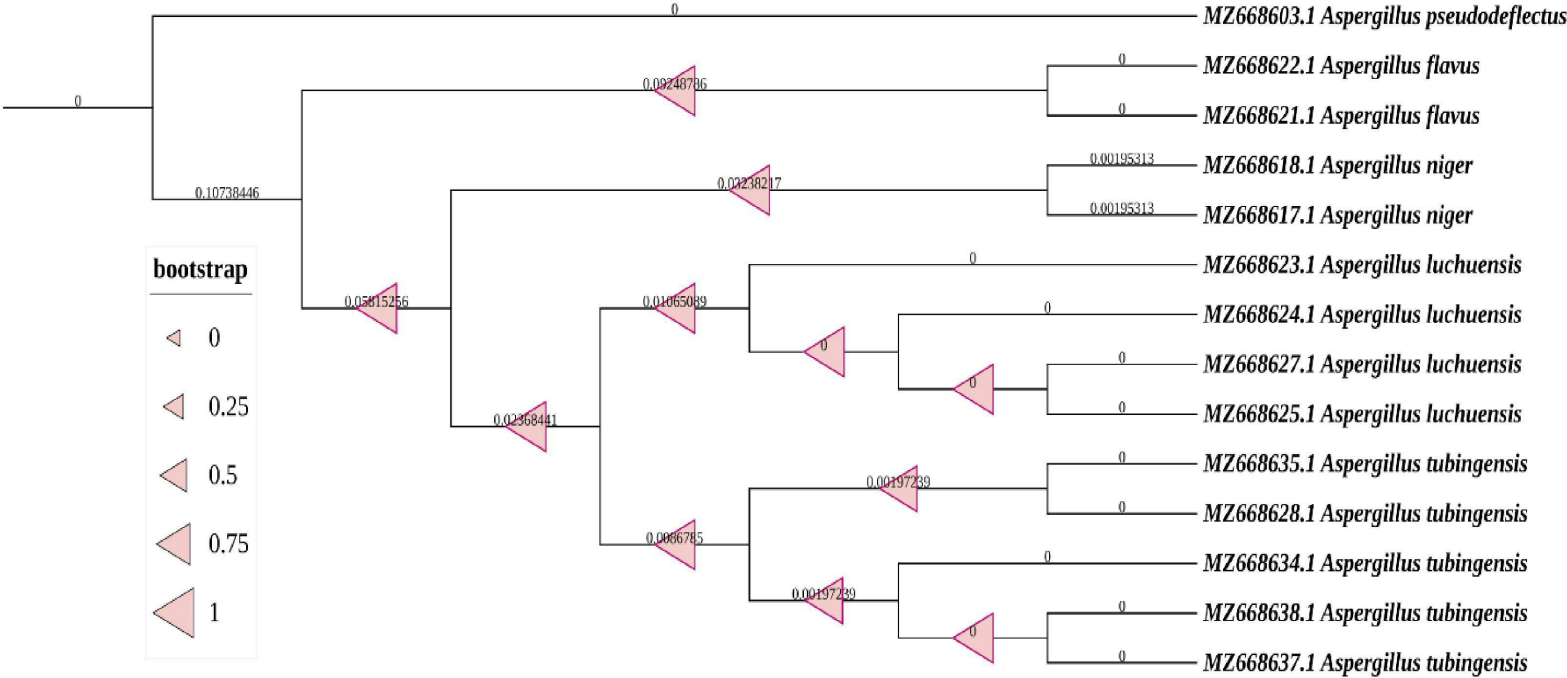
Phylogenetic analysis using the beta-tubulin gene of the isolated *Aspergillus* species.

Of a total of 400 samples, 248 (62%) did not yield fungi. Of the 152 positive samples, 41 samples (27%) presented more than one isolate. The fungi recovered from the hospital environment, including 193 species, are presented in Table 1. Yeast accounted for 40.3% of fungal isolates from hospitals. The most commonly isolated fungal species were 22 *Aspergillus flavus* (11.4%), 21 *C*. *albicans* (10.9%), 17 *Mucor* species (8.8%), 16 *Penicillium* species (8.3%; OM219079), 16 *Candida famata* (8.3%), 15 *Alternaria* species (7.8%; OM756727), 13 *Fusarium* species (6.7%; OM219620 and OM756730), 11 *C*. *parapsilosis* (5.7%), and 10 *Aspergillus niger sensu stricto* (5.2%). *Candida* species were isolated from beds, ventilators, and the tap faucet’s contaminated surface. Mold fungi (*Aspergillus* and *Mucor* species) have been isolated from the floors of the rooms and around the doors, windows, and air conditioners. Figure 1 demonstrates the phylogenetic tree of the *Aspergillus* species isolated from the hospital environment. Most of the strains displayed strong relationships in the bootstrap.

**TABLE 1:** Fungal species isolated from different units of four university hospitals

Mold fungi	Frequency (%)	Yeast fungi	Frequency (%)
*Aspergillus flavus*	22 (11.4%)	*Candida albicans*	21 (10.9%)
*Aspergillus niger sensu stricto*	10 (5.2%)	*Candida famata*	16 (8.3%)
*Aspergillus fumigatus*	4 (2.1%)	*Candida glabrata*	12 (6.2%)
*Aspergillus pseudodeflectus*	1 (0.5%)	*Candida guilliermondii*	6 (3.1%)
*Mucor* species	17 (8.8%)	*Candida parapsilosis*	11 (5.7%)
*Fusarium* species	13 (6.7%)	*Candida tropicalis*	1 (0.5%)
*Acremonium* spp.	2 (1%)	*Candida krusei*	1 (0.5%)
*Acremonium fusidioides*	2 (1%)	*Candida kefyr*	4 (2.1%)
Black fungi	3 (1.6%)	*Filobasidium magnum*	2 (1%)
*Epicoccum* species	3 (1.6%)	*Naganishia albida*	2 (1%)
*Alternaria* species	14 (7.4%)	*Naganishia adeliensis*	1 (0.5%)
*Alternaria conjunctiva*	1 (0.5%)	*Naganishia diffluens*	1 (0.5%)
*Penicillium* species	16 (8.3%)		
*Chrysosporium* species	1 (0.5%)		
*Scopulariopsis* species	6 (3.1%)		
Total (193, 100%)	115 (59.7%)		78 (40.3%)

The susceptibility patterns of isolated fungi are exhibited in Table 2. The *Aspergillus* species isolated expressed relatively low susceptibility to ITR and POS, with MIC90 values of 8 µg/mL. The MIC90 value in CAS in *Aspergillus* species was 0.064 µg/mL, but this value in filamentous fungi other than *Aspergillus* was 8 µg/mL in *Mucor* species, 16 µg/mL in hyaline hyphomycetes (*Fusarium* and *Acremonium* species), 8 µg/mL in *Scopulariopsis* species, and 4 µg/mL in *Penicillium* species. Voriconazole MIC50 value in *Aspergillus* species (0.5 µg/mL) was lower than that of hyaline hyphomycetes (2 µg/mL), *Mucor* species (4 µg/mL), and *Scopulariopsis* species (4 µg/mL). Dematiaceous fungi are most sensitive to CAS (MEC = 0.5 µg/mL) and LUL (MIC90 = 0.5 µg/mL). Hyaline hyphomycetes and *Mucor* species exhibited high MIC90 values for all antifungal agents. *Penicillium* species were sensitive to all antifungal agents except CAS (MEC90 value was 4 µg/mL). Fluconazole and ITR proved to be less active against *Candida* species, with MIC90 values of 16 µg/mL and 8 µg/mL, respectively. *Candida* species presented low MIC values for ISA (MIC90 = 0.032 µg/mL) and AMB (MIC90 = 0.5 µg/mL). Caspofungin with an MIC90 value of 4 µg/mL was not an effective antifungal agent against environmental *Candida* species.

**TABLE 2:** Comparison of *in vitro* activities of antifungal agents (µg/mL) tested against isolated fungal species by CLSI method

Species	Antifungals	Range	MIC/MEC_50_	MIC/MEC_90_	MIC_GM_
*Aspergillus* species (*n* = 37)	Amphotericin B Caspofungin Voriconazole Itraconazole Posaconazole Luliconazole Isavuconazole	0.032–8 0.016–0.064 0.125–8 0.016–8 0.064–8 0.008–0.25 0.008–0.125	1 0.032 0.5 8 0.25 0.032 0.032	2 0.064 1 8 8 0.25 0.064	0.627 0.027 0.509 4.317 0.570 0.043 0.023
*Candida* species (*n* = 31)	Amphotericin B Caspofungin Voriconazole Fluconazole Itraconazole Posaconazole Luliconazole Isavuconazole	0.016–1 0.016–4 0.016–8 0.25–32 0.016–8 0.016–4 0.008–4 0.008–2	0.125 0.064 0.064 2 0.25 0.25 0.25 0.032	0.5 4 1 16 8 2 4 0.032	0.153 0.128 0.117 2.678 0.437 0.235 0.252 0.064
Hyaline hyphomycetes fungi^*a*^ (*n* = 17)	Amphotericin B Caspofungin Voriconazole Itraconazole Posaconazole Luliconazole Isavuconazole	0.125–8 0.016–312 1–8 0.125–16 1–8 0.008–4 0.032–4	1 8 2 4 4 0.5 1	2 16 8 4 8 2 4	0.922 4.346 2.771 4.520 1.703 0.193 0.784
Dematiaceous fungi^*b*^ (*n* = 21)	Amphotericin B Caspofungin Voriconazole Itraconazole Posaconazole Luliconazole Isavuconazole	0.016–0.5 0.016–32 0.016–8 0.016–8 0.016–2 0.008–0.5 0.008–4	0.125 0.016 0.5 1 0.5 0.008 0.064	2 0.5 1 8 2 0.5 4	0.126 0.043 0.470 1.003 0.243 0.028 0.135
*Mucor* (*n* = 17)	Amphotericin B Caspofungin Voriconazole Itraconazole Posaconazole Luliconazole Isavuconazole	0.016–4 0.016–16 0.5–16 0.25–16 0.125–16 1–16 0.008–0.5	0.25 4 4 8 2 4 0.25	2 8 8 8 8 8 2	0.410 1.451 4.340 5.543 1.920 3.539 0.241
*Penicillium* species (*n* = 16)	Amphotericin B Caspofungin Voriconazole Itraconazole Posaconazole Luliconazole Isavuconazole	0.064–0.125 0.016–8 0.016–0.064 0.25–0.5 0.125–0.5 0.032–1 0.125–1	0.064 0.016 0.064 0.032 0.125 0.5 0.125	0.064 4 0.064 0.064 0.250 1 0.5	0.064 0.015 0.039 0.035 0.136 0.806 0.142

^*a*^ Hyaline hyphomycetes in this study included *Acremonium* and *Fusarium* species.

^*b*^ Dematiaceous fungi in this study included black fungi, *Epicoccum* and *Alternaria* species*,* and *Alternaria conjunctiva*.

## DISCUSSION

Fungi are abundant in our surroundings but few are capable of causing infection in humans. There have been reports on the presence of fungi in public places and hospitals (18 – 20). Exposure to environmental fungi in immunocompromised patients can cause subclinical to severe fungal infections (21, 22). In the present study, the fungi isolated from the environment of university hospitals were evaluated along with their sensitivity patterns investigated. In India, four major clusters of fungal infections in patients were reported: invasive candidiasis, cryptococcosis, invasive aspergillosis, and mucormycosis with a 43.4% (*n* = 110) mortality rate on 30 days (23). Rayens et al. in 2018 reported 666,235 fungal infections with an attributable cost of $6.7 billion in the United States (5). The fungi included *Candida*, *Pneumocystis*, and *Aspergillus* infections accounting for 76.3% of diagnosed fungal infections with 81.1% of associated costs. Risks of mortality were more than twice as high in patients infected with fungi as in those without fungal infections (5). Prigitano et al. reported that fungi were isolated from 12% of ICU environmental surfaces sampled, with molds isolated from 70.8%, mainly *Aspergillus fumigatus*, and yeasts, mainly *C. parapsilosis* and *Candida glabrata*, were isolated from 27.1% of positive samples (20). The fungal species reported in the present study are comparable to those recorded in hospital environments in other studies conducted.

In the present study, filamentous fungi like *Mucor*, *Aspergillus*, *Fusarium*, *Penicillium*, *Scopulariopsis* species, and dematiaceous fungi (*Alternaria* and *Epicoccum*) were isolated from the environment. These fungi are known as saprophytic and found in the soil and decaying organic matter, and can cause nosocomial infections in patients, especially in immunocompromised ones (4). Also, these mycoflora can cause infection in immunocompetent patients (24). Spores enter the human body via inhalation or inoculation of the skin or gastrointestinal mucosa and cause sinopulmonary, pulmonary, and other systemic diseases. These organisms are important fungi causing asthma (19). In a study in Japan, the dust of beddings used in 50 houses was examined for fungal flora. The results showed that the yeasts had the largest isolation rate of mycoflora (isolated from 42/50 houses), followed by *Cladosporium*, *Aspergillus*, and *Alternaria* species (19). In the present study, 59.7% of isolates were mold fungi. Mold fungi are existing in different places (25). Based on the ambient temperature, dryness, and humidity of the air, saprophytic fungi are varying by region.

In a study on environmental surfaces in a tertiary care hospital, the fungi recovered from 62.7% of the swab samples were *Aspergillus* (*A. niger sensu stricto*, 25.9%; *A. flavus*, 17.7%; and *A. fumigatus*, 12.4%), Zygomycetes, and dematiaceous species (1). Abbasi et al. reported different filamentous fungi, *Fusarium*, *Penicillium*, *Paecilomyces* species, and *A. niger sensu stricto*, in the indoor and outdoor spaces of hospitals and different departments (26). *Candida* species are the cause of many severe diseases in the intensive care units of hospitals, and they are one of the most common causes of nosocomial bloodstream infections (4). In the United States, non-*albicans Candida* species particularly *C. glabrata* were reported to cause most cases of candidemia (4). In Iran, 598 *Candida* strains were isolated from clinical samples of 10 tertiary care hospitals, with the most commonly isolated *Candida* species being *C. albicans,* followed by *C. glabrata* and *C. parapsilosis* (25). Isolated strains of yeasts from dust samples in Japan were *Naganishia diffluens* species complex and *Filobasidium magnum* (19). *N. diffluens* were the yeasts often isolated from human skin. From 401 environmental samples from ICU wards, yeasts were growing in 27.1%, mainly on computers (25%) and floors (10.9%), and *C. parapsilosis* (42.8%) and *C. glabrata* (28.6%) were the most isolated species (20). The data reported in this study were consistent with some other studies, i.e., 40.3% of the isolates were yeast fungi.

The sensitivity of yeasts and molds to antifungal agents is different, and species with high MIC values for antifungal agents were reported (25, 27). In a study on fungi isolated from nosocomial infections, increased likelihood of resistance to fluconazole was reported for non-*albicans Candida* species like *C. glabrata* (16%), *Candida krusei* (78%), and *Candida guilliermondii* (11%) (4). Dabas et al. reported high MIC values for azoles in *C. albicans*, *C. glabrata*, *C. parapsilosis*, *A. flavus*, *A. fumigatus*, *Rhizopus microspores*, *Rhizopus arrhizus*, and *Mucor circinelloides*. Also, high MEC values for echinocandins in *A. fumigatus*, *C. glabrata*, *Candida tropicalis*, and *C. guilliermondii* were reported (23). All *Aspergillus* species (54 species) isolated from airs in a study by Panagopoulou et al. exhibited low minimum inhibitory or effective concentrations for AMB, micafungin, anidulafungin, POS, ITR, and VOR (1). In the present study, FLU and ITR were revealed to have less activity against *Candida* species, and many filamentous fungi were resistant to antifungal agents. This finding suggests the need for regular monitoring of clinical microbiological data in each area, which can help better manage and treat patients. The limitation of this study was the limited number of samples. If we could evaluate all parts of the hospitals, the number of isolated species would be higher.

### Conclusion

Fungal infections in hospitals are associated with high mortality and cost. In this study, different species of filamentous fungi and yeast were isolated from the environment of the studied hospitals. Some species were resistant to antifungal drugs. We suggest that the future work concentrates on the relationship between the level of saprophytic fungal contamination in the environment of hospitals and fungal infections in high-risk patients.

## Data Availability

All data analyzed during this study are included in this article. Further inquiries can be directed to the corresponding author.
